# Adding Intranasal to Oral Administration of an Ultra-Rapid Near-Universal Drug Regimen Accelerates Relapse-Free Cure of Tuberculosis in Mice

**DOI:** 10.1093/infdis/jiaf315

**Published:** 2025-06-11

**Authors:** Bai-Yu Lee, Daniel L Clemens, Saša Masleša-Galic, Susana Nava, Chiao-Yueh Lo, Jeffrey I Zink, Marcus A Horwitz

**Affiliations:** Division of Infectious Diseases, Department of Medicine, University of California, Los Angeles, Los Angeles, California, USA; Division of Infectious Diseases, Department of Medicine, University of California, Los Angeles, Los Angeles, California, USA; Division of Infectious Diseases, Department of Medicine, University of California, Los Angeles, Los Angeles, California, USA; Division of Infectious Diseases, Department of Medicine, University of California, Los Angeles, Los Angeles, California, USA; Department of Chemistry and Biochemistry, University of California, Los Angeles, Los Angeles, California, USA; Department of Chemistry and Biochemistry, University of California, Los Angeles, Los Angeles, California, USA; California NanoSystems Institute, University of California, Los Angeles, Los Angeles, California, USA; Division of Infectious Diseases, Department of Medicine, University of California, Los Angeles, Los Angeles, California, USA

**Keywords:** tuberculosis, intranasal therapy, bedaquiline, delamanid, relapse-free cure

## Abstract

We previously identified a potent 4-drug oral regimen comprising clofazimine, bedaquiline, pyrazinamide, and delamanid (parabolic response surface [PRS] regimen V) that dramatically shortens by approximately 85% the treatment duration needed to attain relapse-free cure of tuberculosis in mice compared with the standard regimen. Here, we investigated if intranasal administration of the PRS regimen V antibiotics bedaquiline and delamanid on top of the oral regimen would further accelerate tuberculosis cure in mice. We show that adding the intranasal to oral regimen further lowers lung burden of tubercle bacilli and significantly more rapidly achieves relapse-free cure. Hence, adding inhalational to oral therapy can potentially accelerate cure of tuberculosis.

## Teaser

Addition of intranasal bedaquiline and delamanid to an ultra-short oral tuberculosis drug treatment regimen comprising clofazimine, bedaquiline, pyrazinamide, and delamanid improves treatment efficacy and further shortens time required to achieve relapse-free cure in a mouse model of pulmonary tuberculosis.

Tuberculosis (TB) is an airborne infection transmitted via small droplet nuclei containing the agent *Mycobacterium tuberculosis* (*Mtb*), which primarily affects the lungs. Worldwide, one-quarter of the population is infected with *Mtb* and >10 million people develop active TB every year [1]. The standard of care (standard regimen) involves treatment with 4 drugs—isoniazid (INH), rifampin (RIF), pyrazinamide (PZA), and ethambutol (EMB)—for 2 months followed by treatment with 2 of these drugs (INH and RIF) for an additional 4 months. In 2025, treatment guidelines were updated for both drug-susceptible and drug-resistant TB [2]. For drug-susceptible TB, a shorter 4-month regimen—INH, rifapentine, moxifloxacin, and PZA for 2 months followed by this regimen without PZA for an additional 2 months—is recommended. For multidrug-resistant TB, a new 6-month short-course regimen of 4–5 drugs is recommended, rather than the ≥15-month regimens previously administered. Despite improved standard of care, these treatment courses are still too long and are often associated with serious side effects, compliance issues, and the emergence of drug resistance. Better therapies are imperative to shorten the excessively long treatment course and to combat the global emergence of drug-resistant TB.

Using an artificial intelligence-enabled parabolic response surface (PRS) platform, we have identified several combinations of drugs that dramatically shorten TB treatment duration in rigorous mouse models of pulmonary TB, providing relapse-free cure in as little as 3 weeks compared with 20 weeks for the standard regimen [3–6]. Several of these regimens, including PRS regimen V, consisting of clofazimine (CFZ), bedaquiline (BDQ), PZA, and delamanid (DLM), are appropriate for treating drug-sensitive TB and most cases of drug-resistant TB. Based on our studies in the mouse TB model, PRS regimen V is currently being investigated for capacity to shorten TB treatment in a clinical trial of drug-sensitive TB in Haiti and South Africa (ClinicalTrials.gov identifier NCT05556746).

Anti-TB drugs are normally taken orally and thus subject to metabolism in the gastrointestinal tract and first-pass hepatic metabolism before passing into the systemic circulation and reaching the pulmonary lesions. Other noninvasive routes of administration, such as inhalation or intranasal instillation, are attractive as the drugs are delivered via the airway into the lung or absorbed directly into the bloodstream through the nose, avoiding gastrointestinal destruction and hepatic first-pass metabolism.

In this study, we investigate whether the time needed to treat TB in the mouse model can be reduced even further by supplementing oral delivery of PRS regimen V with intranasal administration of 2 of this regimen's drugs—BDQ and DLM. As proof of concept, we demonstrate that combining intranasal treatment with oral therapy can reduce lung burden and shorten the time to achieve relapse-free cure.

## METHODS

### Aerosol Infection of Mice With *Mtb*

All animal studies were approved by and conducted according to the procedures set forth by the University of California, Los Angeles Animal Research Committee. Female BALB/cJ mice at 9 weeks of age were exposed to an aerosolized suspension of *Mtb* strain Erdman in phosphate-buffered saline (PBS) (day 0) as described elsewhere [3]. Two to 3 mice were euthanized 1 day after aerosolization (day 1) to determine the number of bacilli delivered to the lung and an additional 3–5 mice were euthanized 2 weeks after infection (day 14) to determine the lung burden of *Mtb* at the start of treatment.

### Treatment of Mice With Drug Regimens

**Figure 1.**
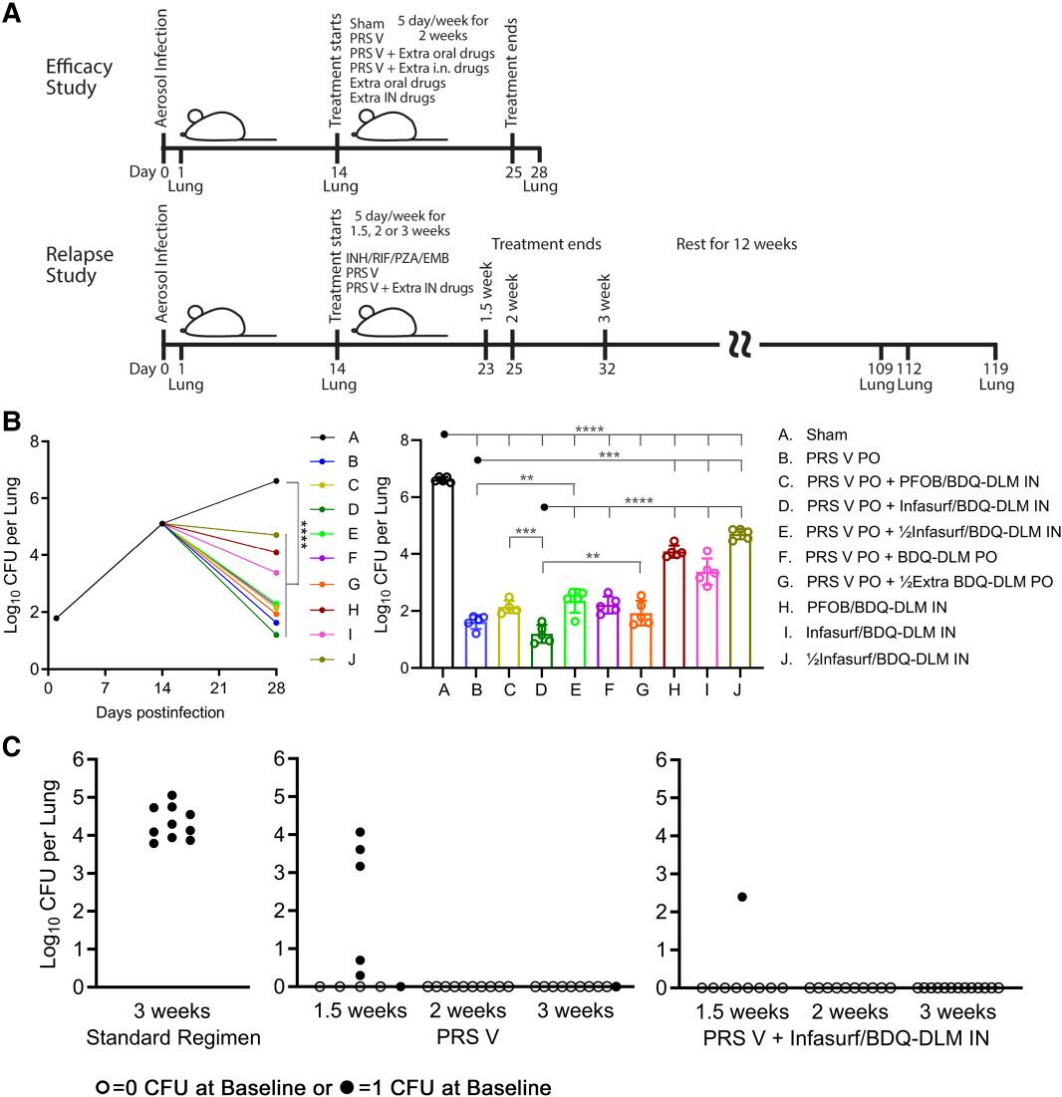
Adding intranasal instillation of bedaquiline (BDQ) and delamanid (DLM) in Infasurf to oral administration of parabolic response surface (PRS) regimen V improves treatment outcome and significantly accelerates relapse-free cure. *A*, Experimental timeline of the efficacy and relapse studies. *B*, Efficacy study (5 mice per group) showing lung burden of *Mycobacterium tuberculosis* over the course of treatment (left panel) and after completion of 2 weeks of treatment (right panel). Data are mean ± standard deviation. *C*, Relapse study (10 mice per time point per treatment group, and 9 and 13 mice for PRS regimen V + Infasurf/BDQ-DLM intranasal treatment group at 1.5 weeks and 3 weeks, respectively) showing lung burden of *M tuberculosis* at 3 months after cessation of therapy. One experiment each was performed for the efficacy and relapse studies. ****P < .0001, ***P < .001, **P < .01. Abbreviations: BDQ, bedaquiline; CFU, colony-forming units; DLM, delamanid; EMB, ethambutol; IN, intranasal; INH, isoniazid; PFOB, perfluorooctyl bromide; PO, oral; PRS V, parabolic response surface regimen V; PZA, pyrazinamide; RIF, rifampin.

Antibiotic treatment was started 2 weeks after aerosol infection (day 14) for 5 days per week (Monday–Friday) for a duration of 2 weeks (efficacy study) or 1.5–3 weeks (relapse study) (Figure 1*A*). The standard regimen consisted of INH, RIF, EMB, and PZA at 25, 10, 100, and 150 mg/kg, respectively. PRS regimen V comprised CFZ, BDQ, DLM, and PZA at 25, 40, 0.83, and 185 mg/kg, respectively. Both standard regimen and PRS regimen V drugs were suspended in 0.15% agarose and administered by oral gavage as described previously [3]. RIF and CFZ were administered separately from the other 3 antibiotics in the standard regimen and PRS regimen V, respectively, with 1 hour between gavages. The supplementary intranasal regimen comprised BDQ and DLM suspended in perfluorooctyl bromide (perflubron, PFOB) or Infasurf at 40 and 0.83 mg/mL, respectively, and was sonicated to produce an emulsion. Mice were first anesthetized with ketamine and then a 20-μL emulsion containing BDQ and DLM in PFOB or Infasurf was administered via intranasal instillation. Sham-treated mice were given 0.15% agarose suspension by oral gavage.

### Assessment of Treatment Efficacy and Relapse

A total of 55 and 81 mice were used in the efficacy and relapse studies, respectively. Mice were euthanized 3 days after the last treatment (efficacy study) or at the end of a 3-month posttreatment holding period (relapse study). Entire lungs were aseptically removed and homogenized in PBS, and colony-forming units (CFUs) in the entire lungs were enumerated as described previously [5].

## RESULTS

### Selection of Vehicles for Intranasal Drug Administration

Delivery of drugs by the intranasal or inhalational route in mice has been accomplished by dry powder inhalation and aerosolization of drug suspensions [7] and intranasal instillation of drug suspensions [8]. Intratracheal instillation or insufflation has the advantage of ensuring efficient delivery into the lungs, but the procedure is time consuming and not well suited for repeated daily treatments of large numbers of mice. Intranasal instillation of drug suspensions, on the other hand, is rapid, noninvasive, and practicable for experiments involving repeated daily treatments of large numbers of mice.

The drugs of PRS regimen V (CFZ, BDQ, PZA, and DLM) have very limited solubility in water, thereby necessitating the use of drug suspension formulations for intranasal administration. For proof of concept, considering the relatively small volume that can be administered by intranasal instillation, we chose BDQ and DLM because of their extremely low minimum inhibitory concentrations compared with PZA and CFZ. As our vehicle for suspending drugs, we chose 2 nontoxic, biocompatible fluids that have been used therapeutically in infants for treatment of respiratory distress syndrome: PFOB [9] and Infasurf, a suspension of calf lung surfactant extract in physiological saline [10]. We found that BDQ and DLM could be suspended in PFOB and Infasurf by sonication and that, although phase separation occurred over time, the drugs were readily resuspended by resonicating in a water bath sonicator.

### Intranasal Administration of TB Drugs Is Efficacious

We assessed the treatment of efficacies of intranasally administered BDQ and DLM, suspended in either PFOB or Infasurf—either alone or in combination with oral PRS regimen V drugs—in BALB/c mice infected with *Mtb*. The amount and ratio of BDQ and DLM in the intranasal suspensions was prepared so as to be the same as that provided in the oral PRS regimen V. Controls included sham-treated mice, mice treated with oral PRS regimen V alone, and mice administered an increased oral dose equal to the sum of the oral regimen and the amount of BDQ and DLM in the intranasal dose. We monitored the body weight of each mouse weekly (Supplementary Figure 1). In general, mice tolerated the treatments with net gain or loss in weights ranging from +7.5% to −9.3%. In animals receiving both intranasal and oral drugs, the stress of the combination of anesthesia and oral gavage, respectively, likely contributed to weight loss. The weight loss in the combined therapy groups is unlikely to be due to systemic drug toxicity because the group that received additional oral drug (matching the amount given in the combined treatment groups) did not show this weight loss.

We determined the number of tubercle bacilli in the lungs 1 day (day 1) after aerosol infection, at the start of the treatment (day 14), and 3 days after the last treatment (day 28). On day 1 after infection, before any mice were treated, the lung burden of *Mtb* was 1.8 logs. In sham-treated mice, the lung burden of *Mtb* increased to 5.1 logs on day 14, and to 6.6 logs on day 28 (Figure 1*B*, left panel). Compared with sham treatment, treatment with BDQ and DLM in PFOB alone by intranasal instillation starting on day 14 was efficacious and yielded a 2.5-log reduction in bacterial burden in the lungs between day 14 and day 28 (*P* < .0001) (Figure 1*B*, right panel). Treatment with BDQ and DLM in Infasurf was even better, resulting in a 3.2-log reduction in lung burden between day 14 and day 28 (*P* < .0001). Lowering the treatment dose of BDQ and DLM in Infasurf by one-half also showed efficacy with a reduction of lung burden by 1.9 logs over the 2-week treatment period (*P* < .0001). The dose response shown with free drugs in Infasurf suggests that a larger dose of drugs delivered intranasally yields greater efficacy.

Treatment with PRS regimen V delivered orally Monday–Friday for 2 weeks (days 14–28) after infection reduced the number of bacteria in the lungs from 5.1 logs on day 14 to 1.6 logs at day 28, which was 5 logs lower than that of the sham-treated mice at day 28 (Figure 1*B*). Adding intranasal treatment of BDQ and DLM in Infasurf to oral treatment with PRS regimen V reduced lung burden by an additional 0.4 logs, although this difference did not reach statistical significance. In contrast, oral administration of extra BDQ and DLM (the same amount as that in Infasurf) in addition to PRS regimen V negatively impacted treatment efficacy, resulting in a 0.6-log higher level of lung CFUs at day 28 compared with mice treated with the standard oral dose of PRS regimen V. This negative outcome likely reflected the fact that the ratio among drugs in PRS regimen V is optimized; hence, increasing the amount of only 2 of the 4 drugs in PRS regimen V delivered via the same oral route of administration altered the drug ratio, resulting in a negative treatment effect. These results demonstrated that the intranasal route of drug delivery to the site of infection is efficacious and that intranasal drug delivery in Infasurf has the potential to enhance the treatment efficacy of oral PRS regimen V.

### Combined Oral and Intranasal Treatment Significantly Reduces Time to Relapse-Free Cure

As the addition of intranasally administered BDQ and DLM in Infasurf formulation to the oral PRS regimen V provided enhanced efficacy against *Mtb* infection, we next assessed whether the treatment would shorten the duration of treatment needed to achieve relapse-free cure. Two weeks after aerosol infection with *Mtb*, we treated the mice with oral PRS regimen V with or without intranasal administration of BDQ and DLM in Infasurf 5 days a week for 1.5, 2, or 3 weeks. For further comparison, we included a group of 10 mice treated with oral standard regimen for 3 weeks. Mice in all treatment groups were then allowed to rest for 3 months prior to assessing relapse, defined as having ≥1 CFU in the entire lung. In mice treated for 1.5 weeks and held for 3 months, 6 of 10 mice (60%) in the oral PRS regimen V group relapsed (Figure 1*C*, Table 1), whereas only 1 of 9 mice (11%) in the combined intranasal and oral therapy group relapsed, a significant difference (log-rank test *P* = .03). In mice treated for 2 weeks, none treated with PRS regimen V with or without the addition of intranasal BDQ and DLM relapsed at 3 months after cessation of treatment. Similarly, in mice treated for 3 weeks, only 1 of 10 mice (10%) treated with PRS regimen V alone and none (0%) of the 13 mice treated with oral PRS regimen V plus intranasal BDQ and DLM relapsed 3 months after cessation of treatment. In contrast to the very low relapse rate observed in the groups treated for 3 weeks with either oral PRS regimen V alone or together with intranasal BDQ and DLM, 100% of mice treated with the standard regimen relapsed 3 months after treatment cessation (log-rank test *P* = .0001 vs either oral PRS regimen V alone or PRS regimen V with intranasal BDQ and DLM).

**Table 1.** Relapse at 3 Months After Cessation of Therapy^a^

Treatment Duration^b^	Standard Regimen^c^	PRS Regimen V^d^	PRS Regimen V + Infasurf/BDQ-DLM^e^
1.5 weeks	…	6/10 (60%)	1/9 (11%)^f^
2 weeks	…	0/10 (0%)	0/10 (0%)
3 weeks	10/10 (100%)	1/10 (10%)	0/13 (0%)

Abbreviations: BDQ, bedaquiline; DLM, delamanid; PRS, parabolic response surface.

^a^Data shown are number of mice relapsed in group/total number of mice in group (percentage of mice relapsed). Relapse is defined as ≥1 colony-forming unit (CFU) in the entire lung 3 months after treatment cessation.

^b^Treatment began 2 weeks after infection for the duration indicated. Mice were treated 5 days per full week (Monday–Friday). Mice treated for 1.5 weeks received only 3 days treatment the second week (Monday–Wednesday).

^c^Standard regimen was administered by oral gavage. Relapsed mice had a lung burden ranging from 6120 to 114 000 CFUs.

^d^PRS regimen V was administered by oral gavage. Relapsed mice had a lung burden ranging from 1 to 11 845 CFUs at the 1.5-week time point and 1 very small colony at the 3-week time point.

^e^PRS regimen V was administered by oral gavage. Infasurf/BDQ-DLM was administered by intranasal instillation. The 1 relapsed mouse had 250 CFUs in the lung.

^f^One mouse on combined oral and intranasal treatment died after receiving the last treatment due to an adverse response to the combination of anesthesia, intranasal instillation, and oral gavage.

## DISCUSSION

It is well known that effective treatment of TB requires combinations of multiple drugs; these are conventionally delivered by the oral route. Combining pulmonary delivery of drugs with oral therapy has the potential to achieve faster patient sputum conversion, alleviate side effects associated with the systemic treatment of oral drugs, and achieve cure more rapidly. As a proof of concept, we have demonstrated in a mouse model of pulmonary TB that administration of BDQ and DLM via the intranasal route in addition to oral PRS regimen V achieves a better treatment outcome than treating with the oral PRS regimen V alone. This enhancement is not merely due to more of the drugs given to the mice. Indeed, we observed an inverse response between treatment efficacy and additional BDQ and DLM administered orally.

Adding one-half or full amounts of additional BDQ and DLM to the standard PRS regimen V oral regimen increased lung burden from 1.6 logs to 1.9 and 2.2 logs, respectively. As the oral route of administration was used for drug dose ratio optimization of PRS regimen V [3], increasing the dose for only 2 of the 4 drugs altered the drug ratio and thus yielded a suboptimal treatment outcome. On the other hand, addition of 2 drugs by the intranasal route enhanced the efficacy of the oral regimen, probably because it delivered the antibiotics directly to the site of infection. It is noteworthy that adding the full BDQ and DLM dose by the intranasal route appeared to be more effective than adding half the amount, thus emphasizing the importance of PRS mapping to optimize drug ratios in treatment regimens.

In human clinical trials, where relapse can only be appreciated by the reemergence of clinical disease, patients are typically followed for 6–12 months to assess the incidence of relapse. However, such a long follow-up period is probably not necessary in an animal trial such as ours. That is because at the end of a 3-month posttreatment observation period, we euthanize all animals and culture the entire lung of each animal for *Mtb* and we stringently define relapse as the presence of a single bacterium or more in the entire lung. While the presence of residual antibiotic in the lung tissue could result in a false appearance of sterilization, the terminal elimination half-lives of BDQ and DLM in mice are 59 hours and 5.9 hours, respectively [11], such that little or no BDQ or DLM would be expected to remain in the mouse lungs 3 months after the end of treatment. Moreover, our inclusion of 0.4% activated charcoal in our agar plates would further minimize the impact of residual antibiotic on CFU determinations. CFZ has a considerably longer half-life, but its impact on residual CFUs in the lungs would also be minimized by our inclusion of 0.4% activated charcoal in the agar plates and, since the dosing of CFZ was identical for the groups receiving oral or combined oral and intranasal PRS regimen V, residual tissue CFZ cannot account for our observation of accelerated relapse-free cure by addition of intranasal BDQ and DLM. Finally, it is important to note that, in our prior studies of PRS regimen V in mice, even when a few organisms (<10 CFUs) were present at 3 weeks posttreatment, there was no relapse observed 3 months later in the equivalently treated group of mice rested for 3 months and then euthanized and evaluated for relapse [3]. While we believe that absence of any detectable CFUs in the lungs 3 months after the end of treatment correlates with relapse-free cure, it is possible that an animal without a single bacterium in its lungs at 3 months posttreatment might nonetheless have shown relapse with a longer follow-up.

In this study, we prepared BDQ and DLM as suspensions in PFOB and Infasurf for intranasal delivery. Intranasal delivery of these drugs in Infasurf showed greater efficacy than delivery in PFOB both by itself and when added to PRS regimen V. PFOB has been used successfully for partial ventilation of neonates suffering from bronchopulmonary dysplasia [12], and additional clinical trials of PFOB for treatment of neonatal bronchopulmonary dysplasia and respiratory distress syndrome are underway. Infasurf is derived from the natural surfactant in calf lungs and approved by the US Food and Drug Administration for treatment or prevention of respiratory distress syndrome in premature babies [10]. Different formulations would be needed for aerosol administration in humans. Unlike mice, humans can inhale deeply through both the nose and mouth, facilitating drug delivery by this route. It has been shown that spray-dried formulations can achieve a deep and highly dispersible lung delivery in humans [13]. In addition, direct administration of antimicrobials into the respiratory tracts of patients with nosocomial pneumonia has shown enhanced efficacy without toxicity in several clinical trials [14].

Our study does have limitations. First, while we used PFOB and Infasurf as vehicles for suspension and delivery of the anti-TB antibiotics, we cannot rule out the possibility that these vehicles contributed to treatment efficacy, either by direct effects on the bacteria or by favorable impacts on the host pulmonary immune response. Second, it should be noted that there is the possibility of both beneficial and adverse drug–drug interactions at the extremely high concentrations of drugs achieved with direct airway administration that may not be seen at the lower concentrations achieved with systemic administration [15]. Third, although drug studies in the mouse model of pulmonary TB are considered fairly predictable of results in humans, their applicability to humans cannot be guaranteed, and confirmatory studies will be necessary in nonhuman primates and ultimately human clinical trials. Fourth, while we detected no relapse 3 months after cessation of treatment, it is possible that relapse might be observed with a longer follow-up. Nevertheless, our data demonstrate proof of principle that airway delivery of antibiotics, when added to an oral drug regimen, has the potential to achieve more rapid relapse-free cure of TB in humans.

## Supplementary Material

jiaf315_Supplementary_Data

## Supplementary Data

Supplementary materials are available at *The Journal of Infectious Diseases* online (http://jid.oxfordjournals.org/). Supplementary materials consist of data provided by the author that are published to benefit the reader. The posted materials are not copyedited. The contents of all supplementary data are the sole responsibility of the authors. Questions or messages regarding errors should be addressed to the author.

## Notes

***Acknowledgments.*** We thank Kaitlyn Kennedy for excellent technical assistance. We thank Otsuka Pharmaceutical Co. for providing delamanid and Onybiotech for providing Infasurf.

***Author contributions.*** M. A. H. and J. I. Z. supervised the project and secured funding. B.-Y. L., D. L. C., and C.-Y. L. prepared reagents and drug suspensions. M. A. H., B.-Y. L., and D. L. C. designed the animal experiments. B.-Y. L., S. M.-G., and S. N. conducted the animal experiments. M. A.H., B.-Y. L., and D. L. C. wrote the manuscript, and all authors reviewed and approved the manuscript for publication.

***Financial support.*** This work was funded by the Department of Defense Congressionally Directed Medical Research Programs (grant number PR190949 to M. A. H.).
