# Supplementary material for: Adding Intranasal to Oral Administration of an Ultra-Rapid Near-Universal Drug Regimen Accelerates Relapse-Free Cure of Tuberculosis in Mice
Source: J Infect Dis. 2025 Jun 11;232(3):e442–7. doi: 10.1093/infdis/jiaf315 (PMC12455299; doi:10.1093/infdis/jiaf315)
Supplement: jiaf315_Supplementary_Data [file jiaf315_supplementary_data.docx]

**Supplementary Methods and Figure**

Manuscript entitled:

**Adding Intranasal to Oral Administration of an Ultra-rapid Near Universal Drug Regimen Accelerates Relapse-free Cure of Tuberculosis in Mice**

Bai-Yu Lee^a,†^, Daniel L. Clemens^a,†^, Saša Masleša-Galic^a^, Susana Nava^a^, Chiao-Yueh Lo^b^,

Jeffrey I. Zink^b,c^, Marcus A. Horwitz^a,^*

^a^Division of Infectious Diseases, Department of Medicine, University of California

Los Angeles, CHS 32-150, 10833 Le Conte Ave., CA 90095-1688, USA

^b^Department of Chemistry and Biochemistry, University of California, Los Angeles, 3013 Young

Dr. East, CA 90095-1569, USA

^c^California NanoSystems Institute, University of California, Los Angeles, CA 90095-8352, USA

^†^ Contributed equally

*Corresponding author: Marcus A. Horwitz, Division of Infectious Diseases, Department of Medicine, UCLA School of Medicine, CHS 32-150, 10833 LeConte Avenue, Los Angeles, CA 90095-1688; Phone: 310-206-0074; Fax: 310-794-7156; E-mail: mhorwitz@mednet.ucla.edu

**A. Supplementary Methods**

**Mice**

Female BALB/cJ mice at 9 weeks of age were purchased from The Jackson Laboratory. Mice were housed 5 per cage and acclimated 6 days prior to aerosol infection. Food and water were provided ad libitum. Physical appearance of the infected mice was monitored daily for signs of distress and illness. Euthanasia of mice was carried out with CO_2_ asphyxiation. All animal experiments were performed with biosafety level 3 practices in the UCLA animal facility and approved by the UCLA institutional animal care and use committee.

**Aerosol Infection**

Glycerol stocks of *M. tuberculosis* strain Erdman were thawed from -80°C storage for preparing a suspension in 20 ml PBS at a concentration of 1.3 – 1.4 x10^6^ colony forming units (CFU) per ml for use in aerosolization. Mice were exposed to the bacteria aerosolized using a Collison 6-jet nebulizer for 30 min (Day 0) as described [5]. Two (efficacy study) or three (relapse study) mice were euthanized one day after aerosolization (Day 1) to determine the number of bacilli delivered to the lung. An additional three (efficacy study) or five (relapse study) mice were euthanized two weeks after infection (Day 14) to determine the lung burden of *M. tuberculosis* at the start of treatment.

**Drugs**

INH, RIF, EMB, PZA and CFZ were purchased from Sigma. BDQ was purchased from MedChemExpress and DLM was generously provided by Otsuka Pharmaceutical Co. Antibiotics were prepared as DMSO stocks and stored at -30°C. Perfluoro-octyl bromide (PFOB) was purchased from Sigma. Infasurf (calfactant) was generously provided by Onybiotech. The Standard Regimen consists of INH, RIF, EMB, and PZA at 25, 10, 100 and 150 mg/kg, respectively. PRS Regimen V comprises CFZ, BDQ, DLM and PZA at 25, 40, 0.83 and 185 mg/kg, respectively. Intranasal antibiotics were prepared by suspending BDQ and DLM in PFOB or Infasurf at 40 and 0.83 mg/ml, respectively, and sonicating to produce an emulsion. Sonication was performed initially the morning of drug administration with a micro-probe tip sonicator and again, just prior to administration, with a water bath sonicator.

**Treatment**

Oral antibiotic treatment with and without concomitant nasal administration of BDQ and DLM was started 2 weeks after aerosol infection (Day 14) for 5 days per week (Monday – Friday) for a duration of 2 weeks (efficacy study) or 1.5 – 3 weeks (relapse study). Both Standard Regimen and PRS Regimen V drugs were suspended in 0.15% agarose and administered by oral gavage as described [5]. RIF and CFZ were administered separately from the other three antibiotics in the Standard Regimen and PRS Regimen V, respectively, with 1 h between gavages. Sham-treated mice were given 0.15% agarose suspension by oral gavage. For intranasal treatment, mice were first anesthetized with ketamine and then a 20 μl emulsion containing BDQ and DLM in PFOB or Infasurf was administered via intranasal instillation.

**Assessment of treatment efficacy and relapse**

Mice were euthanized 3 days after the last treatment (efficacy study) or at the end of a 3-month holding period (relapse study). Entire lungs were aseptically removed and homogenized in PBS. The homogenates were plated on 7H11 agar containing 0.4% charcoal, ampicillin (12.5 μg/ml), amphotericin B (5 μg/ml) and polymyxin B (40 U/ml) either after serial dilution or without dilution to quantitate numbers of CFU of *M. tuberculosis* in the whole lungs. The agar plates were incubated at 37°C, 5% CO_2_-95% air for 4 weeks before enumerating the number of colonies on the plates to determine the lung burden of *M. tuberculosis*.

**Statistical analysis**

GraphPad Prism version 10.4.1 was used for graphing and statistical analysis. One-way ANOVA with Dunnett’s multiple comparisons test was used for comparing treatment efficacy (log-CFU values) for treated versus sham-treated groups. Log-rank (Mantel-Cox) test was used for comparing relapse rate of different treatment groups.

**B. Supplementary Figure**

| 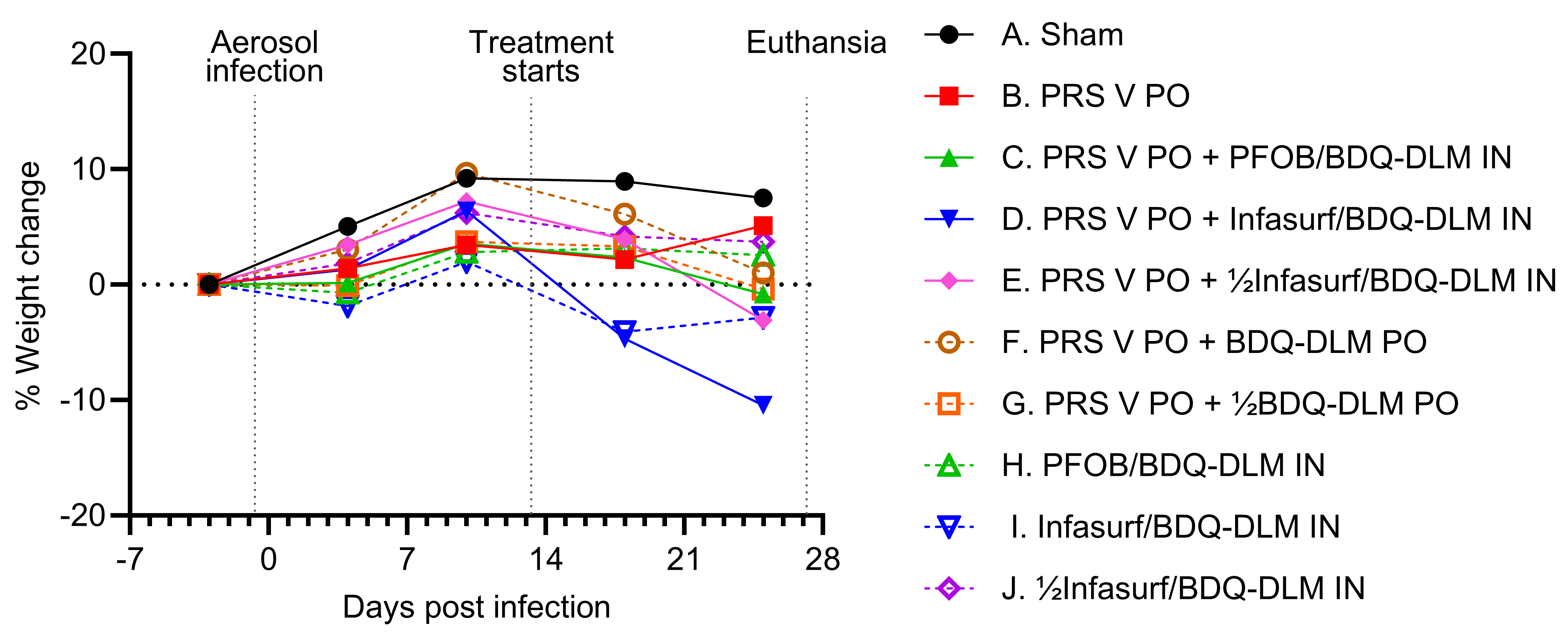 |
| --- |
| **Figure S1.** Weight change over the course of the experiment. The body weight of each mouse was recorded weekly during the experiment. Data shown are mean % weight change of each group. PO, oral administration; IN, intranasal instillation |
